# Veterinarians as Educators

**Published:** 1892-08

**Authors:** 


### VETERINARIANS AS EDUCATORS.

The following circular, issued by the Pennsylvania State Veterinary Medical Association, speaks for itself, and brings up a question which has often been the subject of acrimonious discussion and virulent criticism, especially in those societies where ethics are spread over large sheets of paper, and where they are constantly disregarded by the members :

Pennsylvania State Veterinary Medical Association,

PHILADELPHIA, August 2, 1892.

*To all members of the Pennsylvania State Veterinary Medical Association :*

Through the courtesy of Secretary Thomas J. Edge, of the Board of Agriculture, the members of our association are afforded an opportunity to appear at the various meetings of the Farmer's Institutes which are held throughout every county in the State. This opportunity is one that the officers of this association most heartily commend to all its members. It will bring us more prominently before our people ; it will strengthen our organization, and will be the means oi bringing our profession to a stronger position among the other professions of our State.

We, therefore, as officers do request that every member will, on receipt of this announcement, send in his name to Secretary Ridge, offering to assist in carrying forth this project whenever he may be called upon. It is desirous that in consenting you will name the subject upon which you are willing to prepare a paper. You may be selected to go to most any portion of the State, but it is generally the plan to select a man close to the centre at which the local institute convenes.

Your traveling expenses are provided for by the institute so selecting you A list of those of our members willing to take up this opportuniry must be placed in the hands of Secretary Edge by the 15th of this month. It is, therefore, absolutely necessary that we have in the Secretary's office a list of all those who are willing to take part.

By order of the officers,

W. HORACE HOSKINS, *President.*

W. H. RIDGE, *Secretary.*
Trevose, Bucks Co., Pa.

We believe that the Pennsylvania Association has commenced a grand scheme, which, if systematically carried out, will do great good, both to the community at large and to the profession in particular.

"A little learning is a dangerous thing," but a little more teaches all learners how little they know and the necessity of either learning more or depending upon the learning of others.

Properly written, popular articles upon veterinary subjects, in the secular journals, and such lectures as are proposed by the Pennsylvania Association for the benefit of the farmer, bring before the reader, or hearer, the scope, extent and value of the veterinary profession, and teach them how infinitesimal and limited is the knowledge which is *only* self-acquired and entirely based upon the result of a few practical cases. Popular teaching shows how the general symptoms of disease may belong to widely different troubles, how the special symptoms, as colic, may be the evidence of diseases of opposite character and how these symptoms at first indicate nothing but the presence of a disturbance in the health, while they are positive forerunners of possible grave diseases. The thinking layman, when these subjects are properly brought before him, realizes, as he did not before, the complexity of the subject, and learns to appreciate and depend upon the expert veterinarian. We will look with interest to see the result in Pennsylvania, and trust to see the idea followed in other States.

